# Editorial: Lipid Orchestrated Signaling in Physiology and Pathology

**DOI:** 10.3389/fphys.2022.862073

**Published:** 2022-02-25

**Authors:** Anna Maria Giudetti, Pasquale Simeone, Piero Del Boccio, Daniele Vergara

**Affiliations:** ^1^Department of Biological and Environmental Sciences and Technologies, University of Salento, Lecce, Italy; ^2^Center for Advanced Studies and Technology (CAST), “G. d'Annunzio” University of Chieti-Pescara, Chieti, Italy; ^3^Department of Medicine and Aging Science, “G. d'Annunzio” University of Chieti-Pescara, Chieti, Italy; ^4^Department of Pharmacy, “G. d'Annunzio” University of Chieti-Pescara, Chieti, Italy

**Keywords:** lipid, signaling, metabolism, physiology, pathology

Lipids encompass a wide variety of unique molecular species functionally connected to several physiological processes including the structural maintenance of membrane integrity, metabolism and cell signalingintegrity, metabolism and cell signaling (Wymann and Schneiter, 2008; Holthuis and Menon, 2014). To maintain lipid homeostasis, lipid biosynthesis and uptake are controlled through a complex network of protein kinases and enzymes. Deregulation of lipid homeostasis is at the basis of many pathologies. Thus, understanding the mechanisms of lipid metabolism and signaling control may uncover potential targets for clinical interventions in pathological conditions.

This is well-described in the work of Rudan and Watt that reviewed the compendium of lipid functionality in the context of the mammalian epidermis. As important component of the cell membrane, lipids represent a physical and chemical barrier that protects the epidermis but also function as signaling molecules with a key role in the control of keratinocyte differentiation and in the maintenance of epidermal homeostasis. The authors also emphasized a large-scale approach for studying lipid physiology. A lipidomics-based characterization may accelerate the discovery of novel lipid species that control keratinocyte proliferation and differentiation.

Lipid metabolism is strictly interconnected with signaling. In this scenario, diacylglycerol (DG) is a lipid species at the crossroad between energy metabolism and intracellular signaling. Many enzymes can metabolize DG, including members of a conserved family of lipid kinases named diacylglycerol kinases (DGKs). Nakano and Goto investigated the functional role of DGKε in the lipid-orchestrated pathophysiology of adipose tissues under short-term and long-term high-fat diet (HFD) feeding conditions. In detail, DGKε-KO mice are prone to obesity during early HFD feeding through the regulation of lipid metabolizing enzymes, including adipose triglyceride lipase (ATGL), hormone-sensitive lipase (HSL), and diacylglycerol acyltransferase (DGAT). On the contrary, the authors observed that under long-term (~90 days) HFD feeding conditions, beige adipogenesis is induced in white adipose tissue, which may contribute to enhanced glucose tolerance in DGKε-KO mice. Overall, these data open new questions on the metabolic role of DGK in the regulation of lipid metabolism under early and long-term high fat diet (HFD) feeding conditions. Lipid signaling plays also a role in the induction of many physiological processes including autophagy. In the work of Fernández-Díaz et al., the authors investigated, in pancreatic cancer models, the biological effect of a synthetic lipid, tri-2-hydroxyarachidonein (TGM4), a triacylglycerol mimetic containing three acyl moieties with four double bonds each. TGM4 inhibited proliferation of Mia-PaCa-2 (human pancreatic carcinoma) and PANC-1 (human pancreatic carcinoma of ductal cells) *in vitro* models and *in vivo* xenograft models. Moreover, the cytotoxic effect of TGM4 was associated with elevated reticulum endoplasmic stress and autophagy. The work is of interest because a new synthetic lipid species has been proposed as a potential approach to treat pancreatic cancer.

Several lipid species have a role in the promotion of atherosclerosis. Pokhrel et al. analyzed the effect of Leukotriene D4 (LTD4) on macrophage functions. Interestingly, LTD4 upregulated oxidized low-density lipoprotein receptor-1 (OLR1/LOX-1), and CD36 in a time and dose-dependent manner. Moreover, LTD4 enhanced the secretion of chemokines MCP-1 and MIP1β suggesting that LTD4 contributes to atherosclerosis either through driving foam cell formation, recruitment of immune cells, or both.

Lipids, among which sphingolipids, play a key role in inflammation. The sphingolipid sphingosine-1-phosphate (S1P) pathway in addition to having an anti-apoptotic and proliferative effect has been linked to lung pathology and to SARS-CoV-2 infection. Indeed, the S1P pathway is involved in the aberrant inflammatory process underlying the “cytokine storm” that causes lung injury in COVID-19 patients. Khan et al. reviewed the S1P signaling pathway and the potential clinical implications of a targeted therapy against S1P in COVID-19 and many other diseases involving the S1P pathway (Holthuis and Menon, 2014).

Altered lipid metabolism has emerged as an important player in cancer. Therefore, targeting protein kinases involved in the regulation of lipid metabolism represents an attractive approach to treat cancer. Caglioti et al. by using an electrophysiological approach and noise analysis, investigated the effects of the phosphatidylinositol 3-kinase (PI3K) inhibitors LY294002, and wortammin on the regulation of calcium-activated potassium channel KCa3.1 in glioblastoma models. More in detail, the authors demonstrated the involvement of PI3KC2β isoform, which is LY294002-sensitive and Wortmannin-resistant, on the modulation of KCa3.1 current. These results, coupled with the role of KCa3.1 in radio resistance, could have possible therapeutic implications.

In summary, the results of these studies highlight the role of lipids as bioeffector molecules of several cellular processes with implications for the understanding of human physiology and pathology.

## Author Contributions

All authors listed have made a substantial, direct, and intellectual contribution to the work and approved it for publication.

## Conflict of Interest

The authors declare that the research was conducted in the absence of any commercial or financial relationships that could be construed as a potential conflict of interest.

## Publisher's Note

All claims expressed in this article are solely those of the authors and do not necessarily represent those of their affiliated organizations, or those of the publisher, the editors and the reviewers. Any product that may be evaluated in this article, or claim that may be made by its manufacturer, is not guaranteed or endorsed by the publisher.
